# Pigmented eccrine syringofibroadenocarcinoma simulating malignant melanoma^⋆^

**DOI:** 10.1016/j.abd.2023.03.010

**Published:** 2024-04-08

**Authors:** Bianca Cristina Dantas, Luana Rytholz Castro, Natália Scardua Mariano Alves, Bethânia Cabral Cavalli Swiczar, Neusa Yuriko Sakai Valente

**Affiliations:** Department of Dermatology, Hospital do Servidor Público Estadual, São Paulo, SP, Brazil

Dear Editor,

Eccrine syringofibroadenoma (ESFA) is a rare benign adnexal tumor of eccrine origin, generally found in the extremities of elderly individuals. The first description of ESFA was made by Mascaró in 1963.^1^ They present clinically as solitary or multiple nodules, plaques, or papules of slow growth. On histopathology, ESFA is a benign eccrine ductal proliferation, however there is evidence to suggest carcinomatous transformation in long-lasting lesions.^2^ The present report describes a case of pigmented ESFA with carcinomatous changes without a pre-existing malignant lesion; the histopathological and immunohistochemical studies were essential for the final diagnosis.

An 82-year-old black female patient reported a lesion on the lower lateral aspect of her right leg. Clinically, it appeared as a blackish plaque measuring 4 cm in diameter, with a hyperkeratotic surface and an area with brownish pigment that could be seen in the anterosuperior portion. Dermoscopy showed radiate striae and pseudopods on the periphery of this region (Fig. 1), structures found in melanocytic lesions and commonly associated with malignant melanoma. The lesion had been ongoing for ten years, was painless, and there were no other associated symptoms; nor a history of previous cutaneous malignancy in the region.

**Figure 1:**
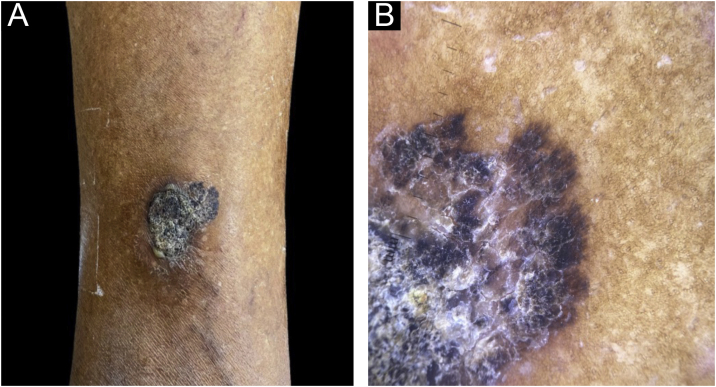
(A) Lower lateral region of the right leg showing a black plaque measuring 4 cm in diameter, presenting a surface and an area with brownish pigment in the anterosuperior portion. (B) Dermoscopy of the anterosuperior region of the lesion showing squamo-crusts on the surface and presence of radiate striae and pseudopodia at the periphery.

Diagnostic hypotheses included malignant melanoma, pigmented basal cell carcinoma, and seborrheic keratosis. An incisional spindle biopsy was chosen due to the size of the lesion. Histopathology revealed the proliferation of basaloid cells forming thin anastomosing cords, connected to the hyperorthokeratotic epidermis, containing lumens and areas of atypia, with sparse pigmented dendritic melanocytes among the atypical basaloid cells (Fig. 2). The melanocytes were easily visible and did not proliferate continously or in nests indicating an associated nevus or melanoma. Immunohistochemistry with Melan-A (A103, mouse monoclonal, Merck KGaATM) revealed sparse dendritic melanocytes among the atypical epithelial cells of the eccrine syringofibroadenocarcinoma (Fig. 3). The basaloid cells were immunostained with EMA, epithelial membrane antigen (E29, mouse monoclonal, Merck KGaA™) and with Ki-67 (SP6, rabbit monoclonal, Merck KGaA™); there was a high proliferative index in groups of atypical basaloid cells within the epidermis. It was also observed that the epithelial cells labeled with EMA were the same ones that exhibited a high proliferative index with Ki-67 (Fig. 4A‒C). Carcinoembryonic antigen, CEA (CEA31, mouse monoclonal, Merck KGaA™) positivity was observed only around the lumens present in the basaloid cell cords (Fig. 4D). The diagnosis of pigmented eccrine syringofibroadenocarcinoma was thus made. The absence of thick cords corroborated the diagnostic exclusion of eccrine porocarcinoma, and the presence of ductal differentiation contributed to the diagnostic exclusion of squamous cell carcinoma.

**Figure 2:**
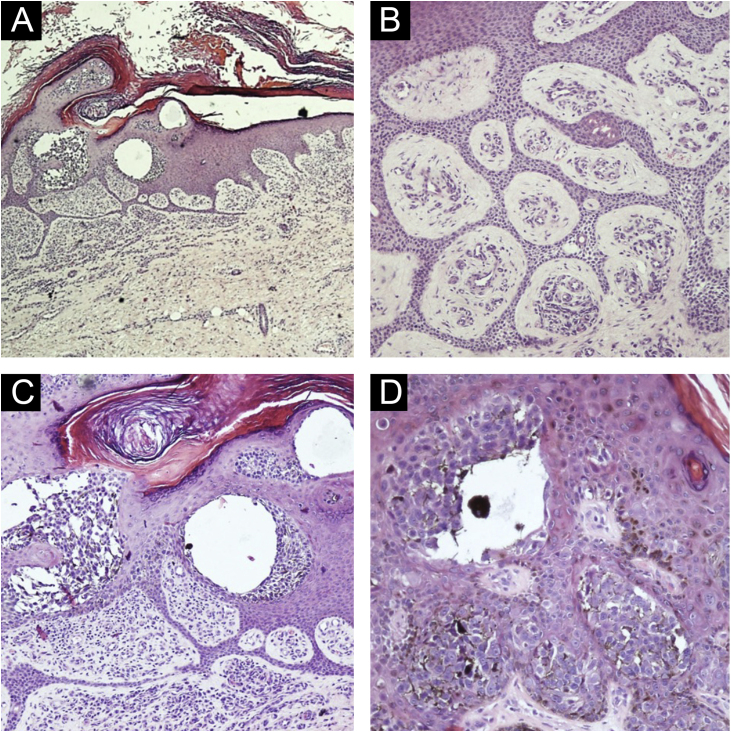
(A) Epidermis with hyperkeratosis, acanthosis, cords of anastomosed basaloid cells and intraepidermal clusters of atypical epithelial cells, (Hematoxylin & eosin, ×40). (B) Epidermis with basaloid cells forming thin anastomosing cords containing lumens and areas of atypia. Stroma with several vessels, (Hematoxylin & eosin, ×200). (C) Atypical basaloid cells within the epidermis, inferiorly forming anastomosing cords, hyperorthokeratosis, (Hematoxylin & eosin, ×200). (D) Atypical basaloid cells within the epidermis surrounded by pigmented dendritic melanocytes, (Hematoxylin & eosin, ×400).

**Figure 3:**
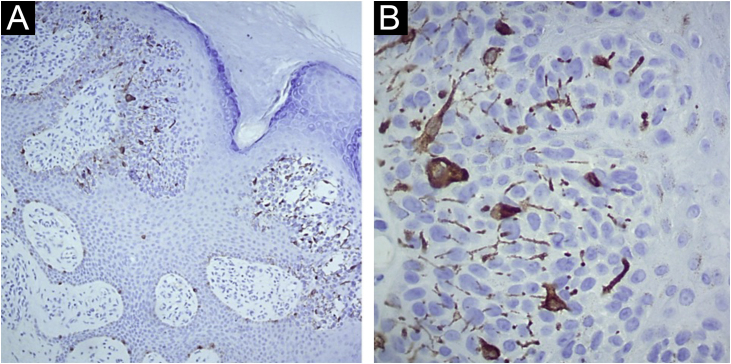
(A) Immunohistochemistry with Melan-A: Immunostaining in sparse dendritic melanocytes among atypical epithelial cells of the eccrine syringofibroadenocarcinoma ×100. (B) Immunohistochemistry with Melan-A: Immunostaining in sparse dendritic melanocytes among the atypical epithelial cells of the eccrine syringofibroadenocarcinoma ×400.

**Figure 4:**
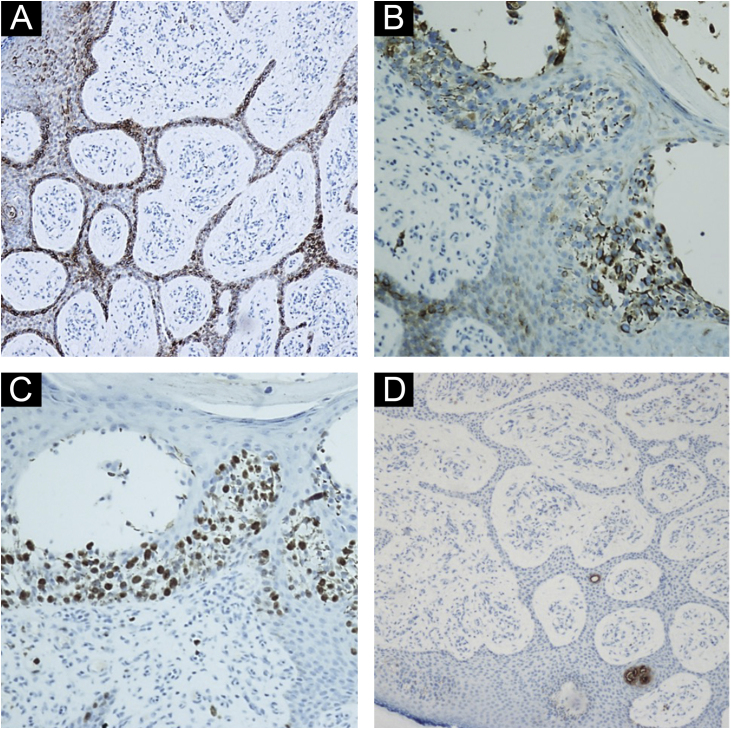
(A) Immunohistochemistry showing positivity with epithelial membrane antigen (EMA) ×200. (B and C) Immunohistochemistry showing that the epithelial cells stained with EMA (composition B) are the same ones that exhibit a high proliferative index by Ki-67 (composition C) ×400. (D) Immunohistochemistry showing positivity with CEA staining in eccrine ducts in the anastomosed epithelial cords ×100.

The lesion was submitted to surgical excision with a 1-cm margin. Sentinel lymph node screening and staging were not performed because it was an *in situ* neoplasm. Surgical excision with complete removal of the lesion is currently the treatment of choice. The histochemical and immunohistochemical analyses of the excised lesion showed in its entirety the same findings found in the incisional biopsy.

ESFA is most commonly found as single, slow-growing plaques or nodules on the extremities of elderly patients. On histopathology, it is represented by thin cords of epithelial cells that anastomose, extending from the epidermis towards the dermis circumscribed by a fibrous stroma, with luminal structures within the epithelial cords. Ductal cells, as well as the cord areas, may show positive expression for CEA. EMA is found diffusely in ductal cells. Its true origin is controversial, but it is believed to be derived from the acrosyringium and eccrine dermal ducts.^3^

In 1997, Starink proposed the ESFA classification into four subtypes: (1) Solitary - non-hereditary, with variable presentation, more prevalent in the middle-aged and the elderly; (2) Associated with Schöpf syndrome - hereditary, with erythematous papules on the palms and soles - Schopf-Schulz-Passarge syndrome (SSPS) is a rare type of ectodermal dysplasia with autosomal recessive inheritance. It is characterized by palmoplantar keratoderma, hypodontia, hypotrichosis, nail dystrophy and multiple periocular and eyelid apocrine hydrocystomas; (3) Multiple lesions not associated with SSPS; (4) Non-familial unilateral linear lesions. Another clinical variant described by Mehregan was the reactive form that develops secondary to inflammatory or bullous dermatoses.

The name syringofibrocarcinoma was first proposed in 1997 by Gonzalez-Serva et al. There are no reports in the literature about metastatic disease or recurrence of eccrine syringofibroadenocarcinoma. Some authors recommend the surgical excision of ESFA to prevent malignant degeneration, although the real risk of carcinomatous transformation is unknown.^4^, ^5^

The mechanisms of ESFA malignant transformation are unclear. In the present case, there was no pre-existing malignant lesion or factors that could increase the risk of malignancy such as radiation or immunosuppression. One must remember that suspected malignancy should always be taken into account in long-lasting skin lesions showing modification and biopsy for histopathology is necessary.

## Financial support

None declared.

## Authors’ contributions

Bianca Cristina Dantas: Design and planning of the study; drafting and editing of the manuscript; collection, analysis and interpretation of data; critical review of the literature.

Luana Rytholz Castro: Design and planning of the study; drafting and editing of the manuscript; collection, analysis and interpretation of data; critical review of the literature.

Natália Scardua Mariano Alves: Design and planning of the study; drafting and editing of the manuscript; collection, analysis and interpretation of data; critical review of the literature.

Bethânia Cabral Cavalli Swiczar: Approval of the final version of the manuscript; effective participation in research orientation; intellectual participation in the propaedeutic and/or therapeutic conduct of the studied cases; critical review of the manuscript.

Neusa Yuriko Sakai Valente: Approval of the final version of the manuscript; effective participation in research orientation; intellectual participation in the propaedeutic and/or therapeutic conduct of the studied cases; critical review of the manuscript.

## Conflicts of interest

None declared.

## References

[1] Hara K., Mizuno E., Nitta Y., Ikeya T. (1992). Acrosyringeal adenomatosis (eccrine syringofibroadenoma of Mascaró). A case report and review of the literature. Am J Dermatopathol.

[2] Pagliuca F., Moscarella E., Argenziano G., Ronchi A., Franco R. (2020). Longstanding eccrine syringofibroadenoma with evidence of carcinomatous transformation. Am J Dermatopathol.

[3] Katane M., Akiyama M., Ohnishi T., Watanabe S., Matsuo I. (2003). Carcinomatous transformation of eccrine syringofibroadenoma. J Cutan Pathol.

[4] González-Serva A., Pró-Rísquez M.A., Oliver M., Caruso M.G. (1997). Syringofibrocarcinoma versus squamous cell carcinoma involving syringofibroadenoma: is there a malignant counterpart of Mascaró’s syringofibroadenoma?. Am J Dermatopathol.

[5] Schadt C.R., Boyd A.S. (2007). Eccrine syringofibroadenoma with co-existent squamous cell carcinoma. J Cutan Pathol.

